# Compatibility of household appliances with DC microgrid for PV systems

**DOI:** 10.1016/j.heliyon.2020.e05699

**Published:** 2020-12-17

**Authors:** Ahmad H. Sabry, Abidaoun H. Shallal, Hayder Salim Hameed, Pin Jern Ker

**Affiliations:** a Institute of Sustainable Energy and Institute of Power Engineering, University Tenaga National (UNITEN), Selangor, 43000, Malaysia; b Department of Electrical Power and Machines, Collage of Engineering, University of Diyala, Iraq

**Keywords:** Electrical engineering, Power system operation, Power converter, Power generation, Power system planning, Household appliances, DC microgrid, Architectures, Power supply, Smart grid, Standardizations

## Abstract

DC distribution of PV systems has spread back especially in the residential sector as a variety of electronic appliances became locally available in the market. The compatibility of household appliances with the best voltage-level in a DC environment is the field that still in the research phase and has not yet made a practically extensive appearance. This paper mainly discusses this issue by providing a review of the concerning research efforts, identifying the gaps in the existing knowledge. The work explains the electrical diagrams of the recently produced appliances, classifying them to get an understanding of how each one consumes energy. It includes exploiting the recent dependence of the commercial appliances on power electronics to improve the efficiency of the existing DC distribution systems by extrapolating new architectures. The proposed topology has a DC distribution environment with two levels of voltage for all appliances. Appliances performances have been evaluated by calculating the energy transfer efficiency. The outcomes of this work can help in designing more efficient DC power distribution networks with minimal energy converters and establishing standardizations for DC microgrids.

## 1 Introduction

Solar photovoltaic (PV) is a DC renewable power source and the present topology integrates such power source to the power distribution infrastructure with the necessity of using DC-AC converters. Producing of electronic household appliances is rapidly increasing in homes and workplaces, which forces to insertion of the external or internal AC-DC converters to link the DC appliances to the AC distribution topology of the existing power systems. Most of the existing on-grid or off-grid battery-based PV systems are still based on AC environment as power distribution for load, while most of the household appliances direct towards electronic-based loads [1]. It is time to think about utilizing the way that electronic-based appliances consume power, especially in PV system applications to save more energy [2]. PV systems with battery storage include DC primary power source, which is the solar PV, backup power source, and the batteries, thus, have already stable DC bus. The inverter in the traditional PV system uses the battery voltage level to generate an AC bus for system loads. Such schemes have many components that expose losses due to the use of many conversion devices even with high-quality equipment. Several studies discussed the energy efficiency of a DC environment in battery-based PV microgrids for homes, but either these studies optimize or improve the energy efficiency through controlling the scheduling of load operation that may not agree with the user desire. This work increases the efficiency of such a system by decreasing the number of energy conversion devices and mainly dispensing the use of DC-AC inverter.

### 1.1 The voltage level of household appliances

This section outlines a review of voltage levels for DC microgrids in residential buildings that lay between a distributed generator and loads relying on practices and existing experience. Due to a lack of standardizations, various voltage levels have been offered in the literature varying from 12V to 800V. Standard voltage levels for DC distribution systems are introduced to reduce system complexity and losses. Although some efforts still try to developing these standards [3, 4], these DC voltage levels may be inspired by the current traditional applications, such as data centers that operating with 380–400 V, telecommunication applications with 48V, transportation camping vehicles with 12–24 V. This work aims, in this section, to get find an appropriate voltage level that fit almost all household appliances based on the previous work and the recent advances in power electronics of appliances, and taking into account energy efficiency, user safety, and cost-saving. In a DC system, the power loss $\text{Δ}P_{DC}$ and the voltage drop $\text{Δ}V_{DC}$ can be calculated from the load power consumption P which is equivalent to the active power for the ac load [5], $\text{Δ}P_{DC}$ and $\text{Δ}V_{DC}$ can be written as:

(1) $$\text{Δ}P_{DC}=2R\frac{P^{2}}{{V_{DC}}^{2}}$$

(2) $$\text{Δ}V_{DC}=2R\frac{P}{V_{DC}}$$

Where R denotes the resistance at $80^{o}$C.

A summary of the noteworthy contributions on the voltage levels for appliances under DC microgrid systems is listed in Table 1. The existing DC bus levels in residential applications encountered in the literature, with their advantages and disadvantages, are presented in Table 2.

**Table 1:** A summary of noteworthy contributions on voltages levels and the tested appliances for DC microgrid systems.

Year	Reference	The investigated household appliances	Home distribution voltage	DC output voltages	Remarks
2003	[5]	Fluorescent light, PC (with monitor), Fax, Copy-Print machine, Dishwasher, Freezer, Fridge, Exhaust fan, Cooker, Water boiler, Coffee machine, and Microwave oven.	400/230	48, 120, 230, 326, 400	The feasibility of a DC network has been discussed but only in terms of voltage drop in the wiring under different voltage levels.
2013	[6]	Induction Heating Cooktop	380	380	Although authors got significantly higher efficiency when the supply is 380V DC than 230V AC, only induction heating was considered in the study.
2015	[7]	Lights, chargers	280	48	Although authors got a significant reduction in THD when the supply is 48 V DC than 220V AC, A DC-DC converter was used to feed the DC appliances in the study.
2018	[8]	four Variable Voltage and Variable Frequency (VVVF) elevators	380	380	Authors state that the presented hybrid AC with DC power has higher conversion efficiency than a single AC power system, but their study application is on an elevator only.
2016	[9]	Coffee maker, Computer, Energy saving lamp, Fluorescent lamp.	230	230	The author discusses only 4 appliances
2016	[10]	The wet-grinder and the dough-maker for Net-zero energy Homes (NZEH).	240 V AC 120 V DC	5,48,120	Modifications on two types of appliances for better performance with DC.
2017	[11]	Air Conditioner, Microwave Oven, DC Fan, Cloth Washer, Refrigerator, Air cooler, water pump, electronic appliances	308 DC	48	Instead of using universal and induction motors in rotary appliances, BLDC (Brushless DC) motors are proposed for 48V DC. With a DC-DC converter.
2017	[12]	Variable Speed Drives (VSD) air-conditioner	230	230	Although the authors discuss the efficiency of AC and DC in residential power distribution systems, they considered only the losses of power transfer through the wiring
2018	[13]	DC Fan, Mobile charger, Laptop, LED light	240	48, 5, 19, 72	Although authors got significantly higher efficiency when the supply is DC than 240V AC, A DC-DC converter was used to feed the DC appliances in the study.
2018	[14]	Lights, Phone charger, Laptop, Water heater, Streets lights, Fluorescent tube, TV, Decoder, Shaver	24V	230	The authors used the Boost converter from 24V DC to 230V DC.
2019	[15]	Space Heating, Water Heating, Space Cooling, Lighting, Refrigeration, Electronics, Wet Cleaning, Cooking, Computers.	240 V AC	24, 48, 380 and±170 V	Only an overview of the recent efforts in the field of efficiency/energy for the DC power distribution system.

**Table 2:** Comparison of existing bus level for residential application.

Voltage level	Advantages	Inconveniences	Application domain in residential
12V	Safe level to deal with. DC appliances are commercially available with this level	Its losses become high as the transferred power gets higher. Designed for a relatively low load of power usage	Low load application, short distances.
24V	Within the standard of EMerge Alliance, which is a safe a low DC level power distribution		Higher power and distance longer energy dispatch than 12V applications
48V	Within the standard of IEEE standard for a DC microgrid in remote and rural electrifications. DC appliances are commercially available with this level It is required simple protections		Communications and networking instruments, and off-grid PV home systems
380–400V	Within the standard of EMerge Alliance of buildings Ease compatibility with utility voltage 230 VAC (It is possible to fit some AC loads directly on DC and minor modifications may be required for others). More appropriate for DC or AC distribution systems in domestic applications.	Step-down converters are required for adapting with most of the existing locally available appliances. Protection is compulsory	Microgrid with grid-connected possibility. Commercial and residential buildings
1500V (+750V)	Appropriate for high power transmission		Industrial applications, commercial and aggregated buildings

From the related literature listed in the above tables, several voltage levels of DC have been recommended, such as 48, 120, 220/230, and 311/326 V. For 48V, such a voltage is considered inherently safe and does not require any protection. It is already used in the market in some devices like data centers, telecommunications, and PC networking, but this level becomes infeasible at high power consumption devices due to wiring losses. For 120 V, no protection is required against indirect contacts, but there are no 120V appliances available in the market for countries with 220/230 V electricity. For 230 V, this voltage allows the use of resistance appliances, such as incandescent lights and heating, with no changes, since it has a similar RMS value for the 230 V AC. For 311/326 V, this value reflects the peak value of the voltage 230 V AC, where most recent appliances have an input diode rectifier, which enables them to be operated under this DC level. A few modifications may be preferred by just removing that rectifier when more efficiency is required, but it is not necessary since it protects against reverse polarity.

### 1.2 Protection

The protection of a DC system is a big challenge, as DC has no natural zero points. 3-phase AC circuit breakers (CBs) were suggested by connecting the three contacts in series as a DC protection to remove the spark [16]. Besides, some articles discussed DC short-circuit problems [17]. A protection circuit for a low voltage DC microgrid proposed in [18], where different faults at different locations on the DC grid have been addressed, the results demonstrated that the commercial AC protection elements are possible to use with DC, such as CBs and fuses to protect loads and batteries. In the same context, converter circuits with IGBT modules are more sensitive to over current and require faster protections that can be represented by a hybrid ultra-fast DC CBs. It was also revealed that protection problems might arise when faults of high-impedance ground occur. Authors in [19] proposed a low-cost procedure to indicate and isolate fault for a multi-terminal DC microgrid via fast DC switches as an alternative of CBs. This method was relied on extinguishing faults of the DC through disconnecting all AC CBs.

The current arcing problem during the unplugging of household appliances in the DC system was discussed in [20]. Their method is to add a shunt diode and capacitor branches with the plug. This method has been modified in [21], where these components were included within the electronic circuit of converters in the system to interrupt the current faults. It was presented that the DC system can be fast detected and isolated by combining the converters by associating relays and by using overcurrent protection criteria for those relays. Furthermore [22], and [23] compare mechanical and electronic CBs of 400V DC system, and they were shown that electronic CBs perform better than mechanical ones concerning current limitations, remote controllability, trip time curve adjustment, rated current controllability, wire break indication, monitoring functions for voltage and current. In [24], the author presented a DC hybrid CB with very fast contact opening and Integrated Gate Commutated Thyristor (IGCT), which comprises the fast-speed switching and bi-directional features.

Based on the above literature survey, this work considers two levels of voltages for the proposed DC environment, 220–230 V, and 300–312 V DC. The 230 V voltages allow the use of resistance appliances, such as incandescent lights and heating, with no changes, since it has a similar RMS value for the 230 V AC. The voltage level 311/326 V, which represents the peak equivalent value of the voltage 230 V AC, is selected since most recent non-resistive appliances have an input diode rectifier, which enables them to be operated under this DC level. Removing a rectifier circuit for additional efficiency might not necessary as it protects against reverse polarity. This paper mainly discusses the compatibility of household appliances with the best voltage-level in the DC environment though explaining the electrical diagrams of the recently produced appliances, classifying them to get an understanding of how each one consumes the energy. It includes exploiting the recent dependence of the commercial appliances on power electronics to improve the efficiency of the existing DC distribution systems by extrapolating new architectures. The work also proposes a topology with a DC distribution environment that has two levels of voltage for all appliances. The appliances' performances have been evaluated by calculating the energy transfer efficiency.

## 2 Proposed configuration

**Figure 1:**
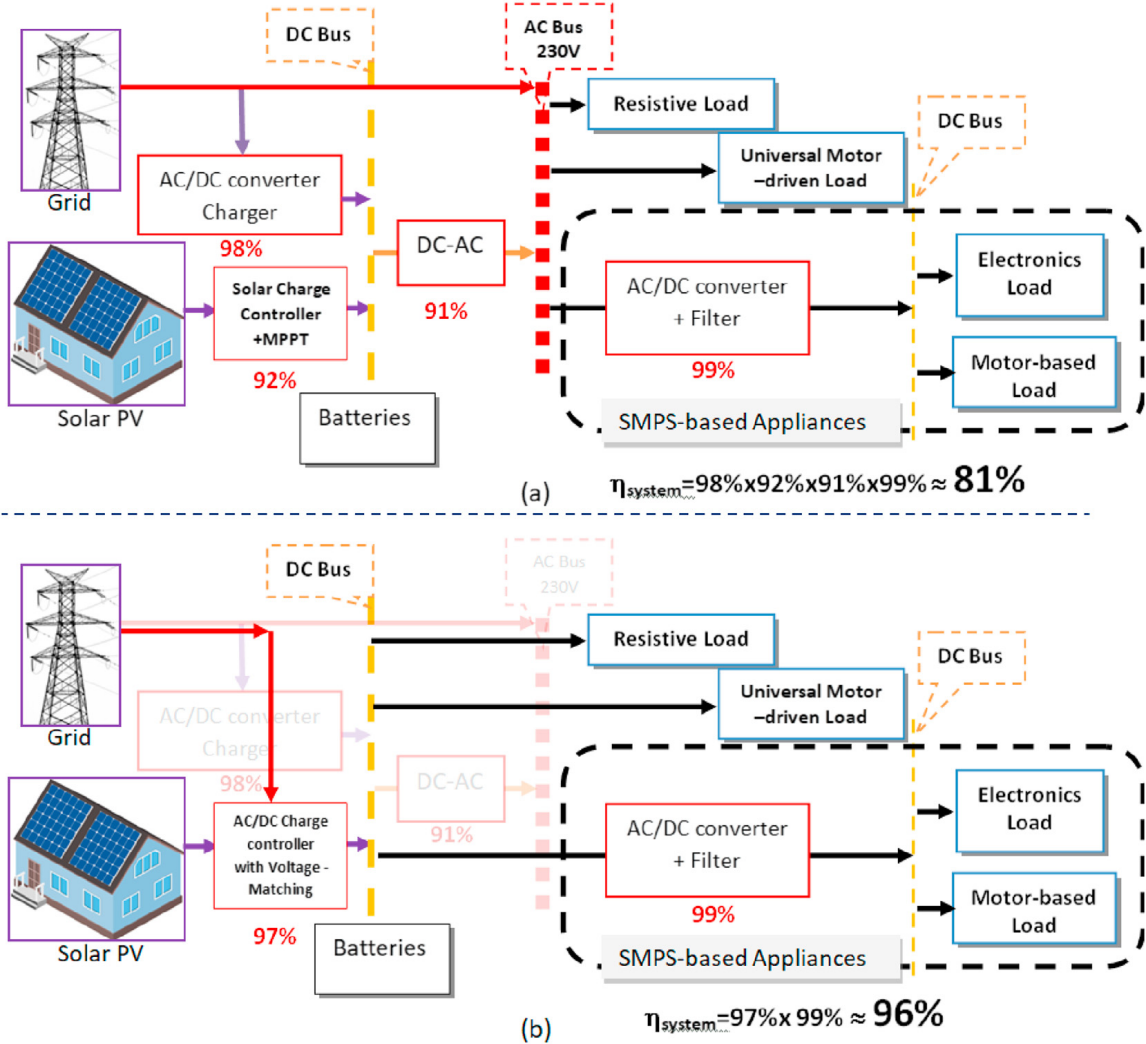
Topologies of ON-grid PV home microgrid with storage batteries: (a) traditional with AC distribution, (b) the proposed configuration with DC distribution, and no inverters.

The proposed topology considers the efficiency values of each stage that have been specified according to the corresponding researches in the previous related work [10, 14, 25, 26, 27, 28, 29, 30, 31, 32]. Based on the system efficiency values, a traditional ON-grid PV home microgrid system and the proposed one is shown comparatively as in Figure 1.

For the traditional ON-grid PV home microgrid system, Figure 1(a) shows about 81% cumulative efficiency, which is due to the existence of four main energy converters in the system. Therefore, the main objectives of the proposed system are to overcome 1) the loss issue of power conversion devices of the traditional AC systems 2) the multiple voltage levels, and, 3) the limitations of using locally existing appliances of the existing DC systems. The proposed configuration relies on the recent advances in household appliances that are electronics-based loads in consuming electrical power when functioning. A voltage-matching between a source and a load is the concept adopted to overcome these issues by setting the battery bank of the DC-bus such that its fully-charged voltage ($V_{FC}$) being equivalent to the PV voltage at MPP ($V_{mp}$) and also equivalent to the DC value of the 230 V AC of the utility grid. This battery system provides an auxiliary/tap voltage, which is necessary for the resistive appliances category. This voltage level is called ($V_{FCR}$), which is equivalent to the active power of the 230V AC, thus 230V DC. To select automatically between $V_{FC}$ and $V_{FCR}$ when resistive appliances being connected, a recognition circuit to extinguish a resistive load is a crucial issue. The proposed topology with its expected efficiency is shown in Figure 1(b). The corresponding researches in the previous related work that this study based on to design the diagram is listed in Table 3.

**Table 3:** Minimum and Maximum efficiency values for the conversion devices of PV home system.

Converter name	Min μ	Max μ	Source
DC-AC	0.84	0.99	[32, 33]
AC-DC	0.91	0.95	[32, 34, 35]
DC-DC	0.82	0.95	[32, 36, 37]
Solar-Battery charger	0.95	0.95	[32, 38]

μ is efficiency.

**Figure 2:**
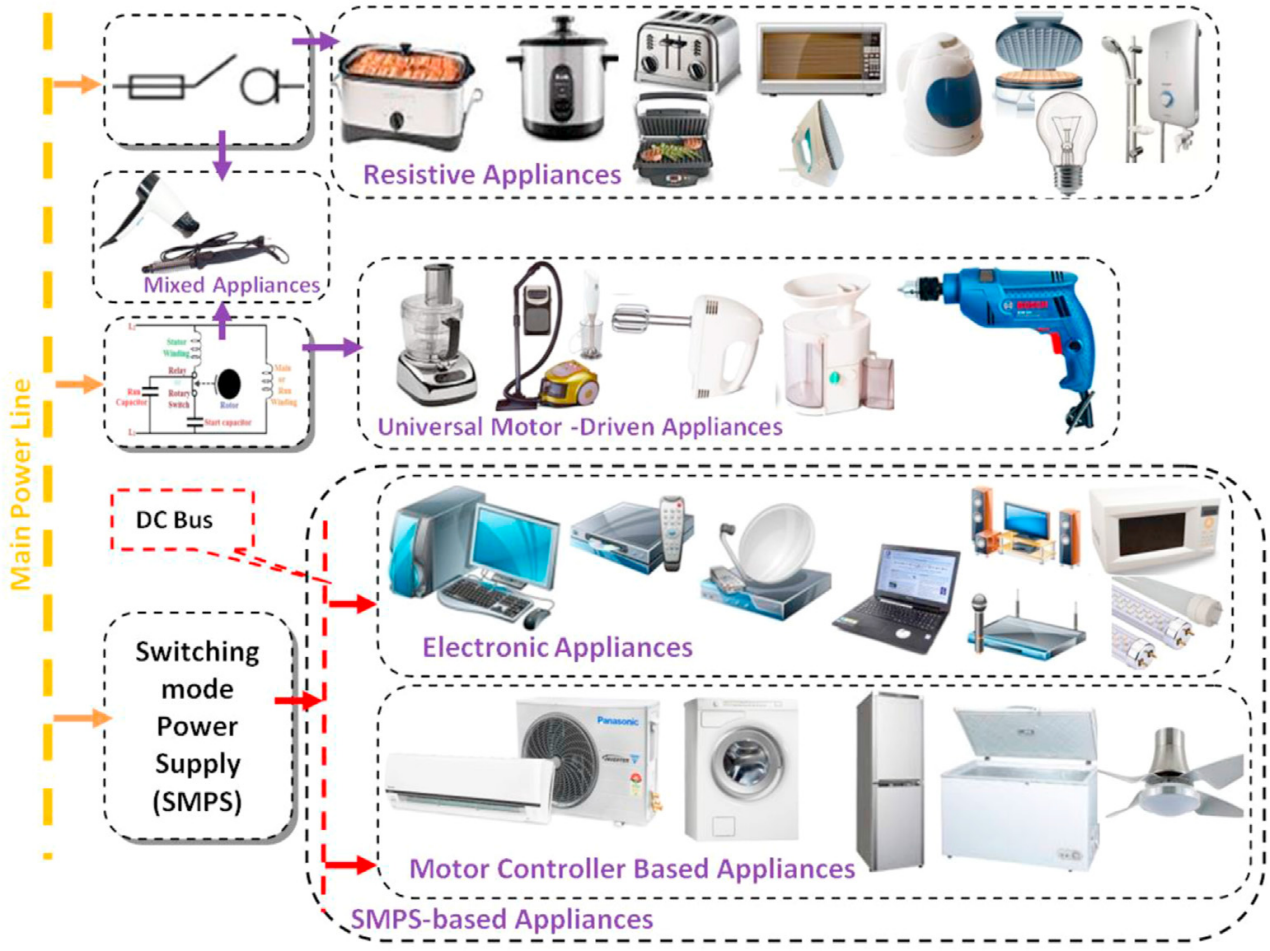
Recent Appliances Classification according to their power consumption and components.

To understand how each category of appliances consumes the power, the appliances have been classified according to the compatibility with a DC source. The classification criteria mainly depend on related publications, practical experiences, and datasheets of appliances. The required energy measurements such as voltages, currents, time, and temperatures are collected by employing a data acquisition card with appropriate sampling frequency to capturing the potential power surges that occur with particular appliances’ components. This classification can be demonstrated with the most popular household appliances in Figure 2.

Some appliances mix two categories such as a hairdryer. As mentioned in the introduction section, most household appliances became electronics-based loads that have a common circuit called switching mode power supply (SMPS). The appliances with SMPS rectify the input AC power source into DC power to drive the loads such as motors and mechanical components.

All resistive appliances are possibly connected to DC with no amendments, only a problem becomes visible as an appliance has contact switches inside, because such contacts may produce an arch with DC [28, 39]. Common traditional resistive appliances and their DC-based alternatives are listed in Table 4.

**Table 4:** Common traditional resistive appliances and their DC-based alternatives.

	Traditional appliance	Alternative towards DC compatibility	References
1	coffee maker	Electronic coffee maker	[40]
2	Rice cooker	Electronic Rice Cooker	[41, 42]
3	Electrical stove	induction stoves	[43]
4	incandescent lamps	LED lights	[31]

### 2.1 Universal motor-driven appliances

**Figure 3:**
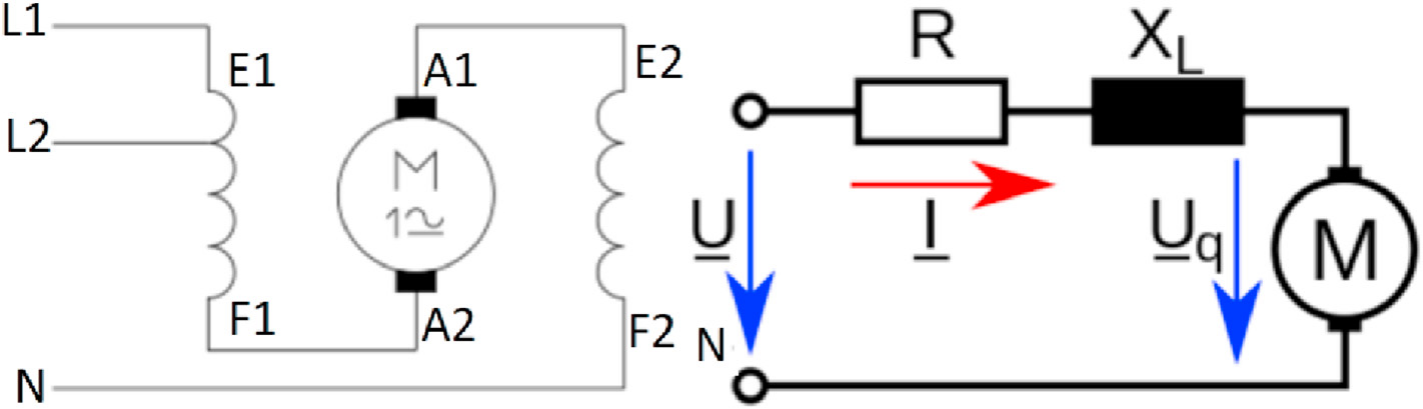
Universal motors' field coils and its equivalent circuit.

The universal motor is the main part of some appliances such as vacuum cleaners, mixers, and some electrical tools like drills and saws [44, 45]. Universal motors are types of electric motors that able to operate on either DC or AC power, its stator magnetic field is created electromagnetically [46, 47]. The motors' field coils and its equivalent circuit are shown in Figure 3.

### 2.2 SMPS-based appliances

**Figure 4:**
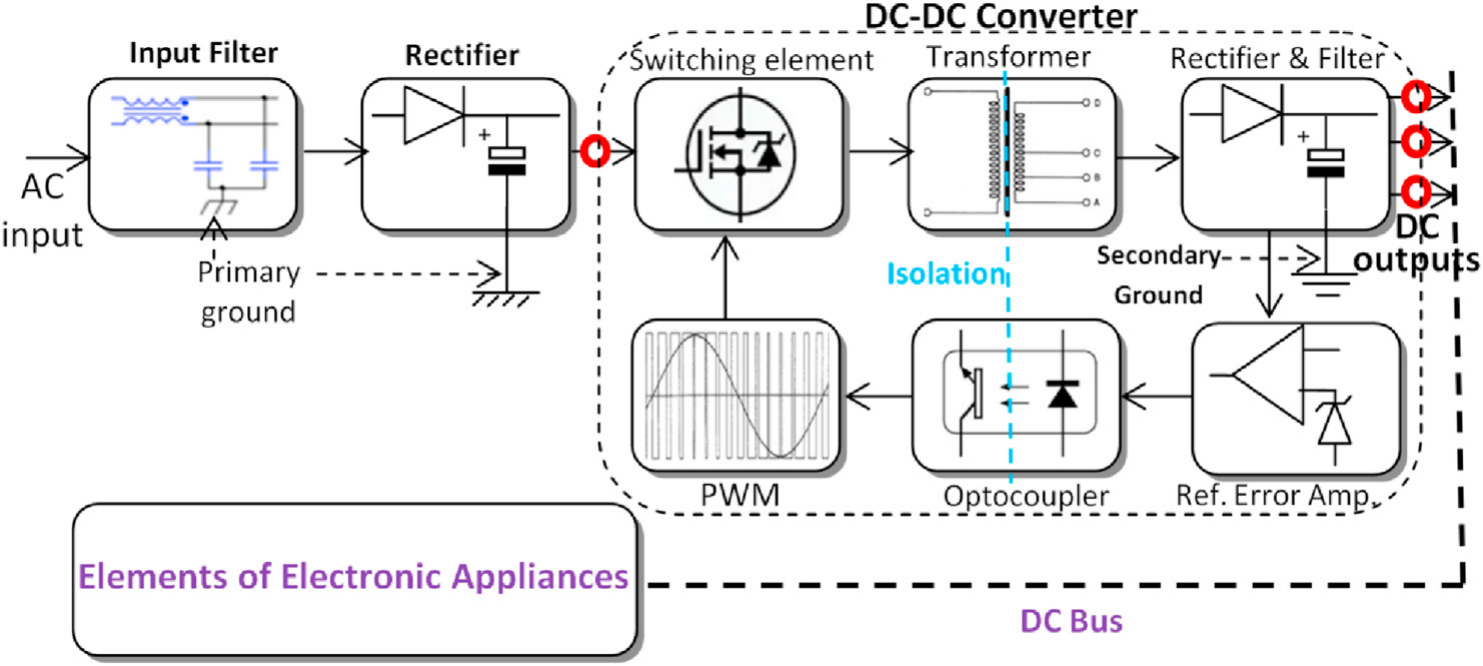
The common power supply for the electronic household appliances. The red circles represent the candidate DC points.

Based on several related studies [8, 47, 48, 49, 50, 51, 52, 53] and the practical check, all the appliances of this category can be functioning on AC and DC power source. A general block diagram, in terms of electrical power stages with SMPS-based appliances, can be described in Figure 4, where the proposed DC connections are marked with red circles.

#### 2.2.1 Compressor-based air-conditioner

**Figure 5:**
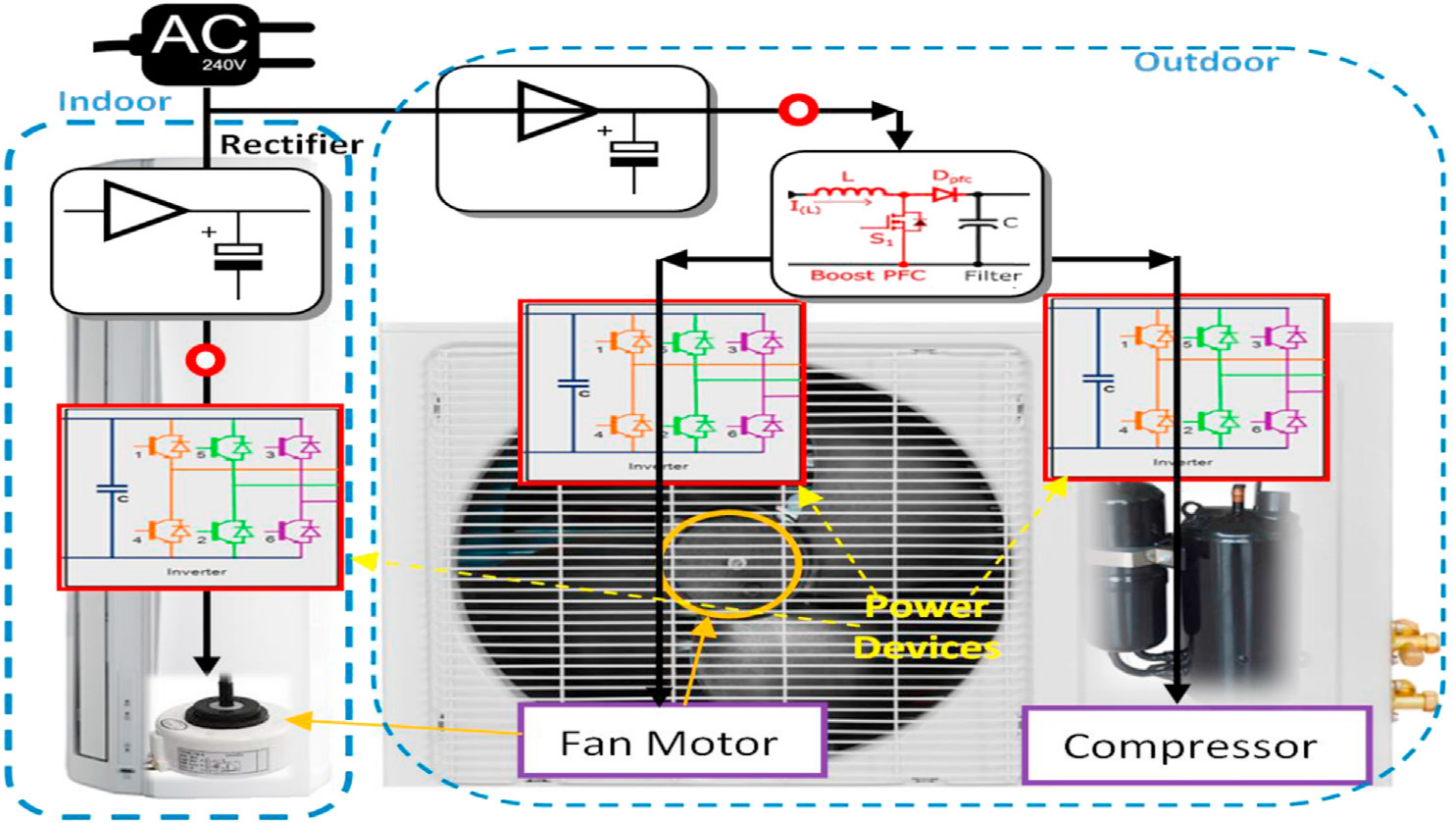
Block diagram of an air-conditioner of a variable-speed compressor.

For traditional air-conditioners or refrigerators, the compressor is composed of an AC induction motor and running with a limited fixed speed. The speed of rotation is a particular multiple of the input frequency. Compressors with fixed-speed produce a high influence on compressor mechanics and the whole temperature control mechanism. In the same context, the new inverter technology-based air-conditioners depend on power electronic components for handling the source electrical energy to drive their equipment. The outcome of the experimental inspections for all these available appliances proves that they are SMPS-based appliances since the SMPS circuit is its input power stage. For that reason, it is possible to operate such type of appliances with a DC supply directly. The block diagram of the inverter driven air-conditioner is demonstrated in Figure 5, where proposed DC connection points are marked with red circles.

**Figure 6:**
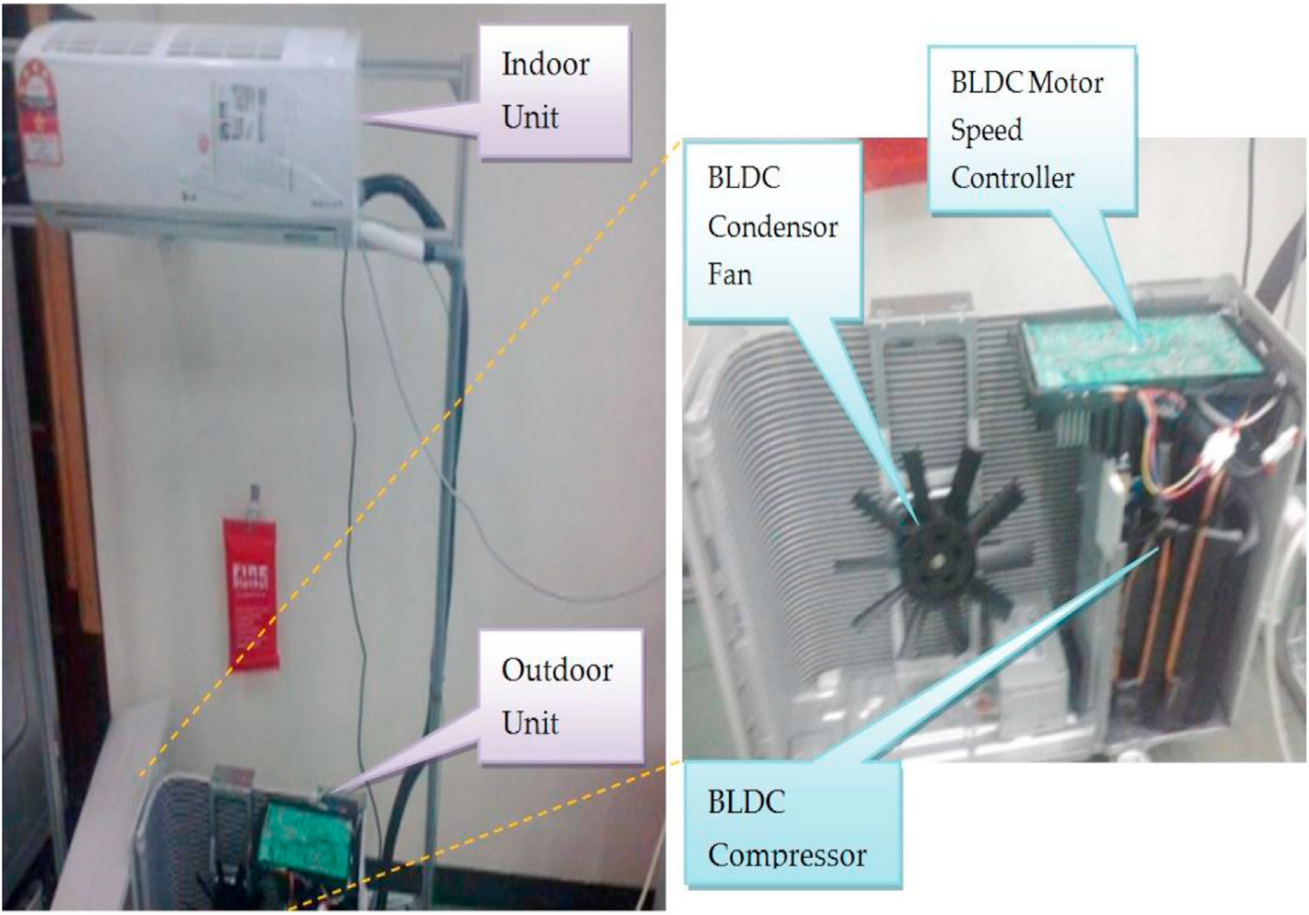
Air-conditioner with variable speed compressor.

The experimentally drown diagram shows that the AC-DC rectifier creates a DC bus of 311 V, as practically measured, which feeds the inverter circuit. The inverter circuit mainly includes insulated-gate bipolar transistors (IGBTs) or MOSFETs to drive the compressor motor with the required power. The controller electronics require auxiliary voltages with DC levels such as the 3.3 or 5, and 12–15 V. These are created as auxiliary voltages by the SMPS circuit for the feedback circuitry and gate drivers. Figure 6 shows the experimental setup and the modification for an LG brand air-conditioner of,1.0 HP, Btu/h = 8600, 3.9 Amp, 220–240 V, 50 Hz [54]. The measurements have been collected via a wireless ZigBee-based monitoring card with appropriate sampling frequency, which is employed to record the currents, voltages, and temperatures required for energy measurements [47].

#### 2.2.2 Compressor-based refrigerator

**Figure 7:**
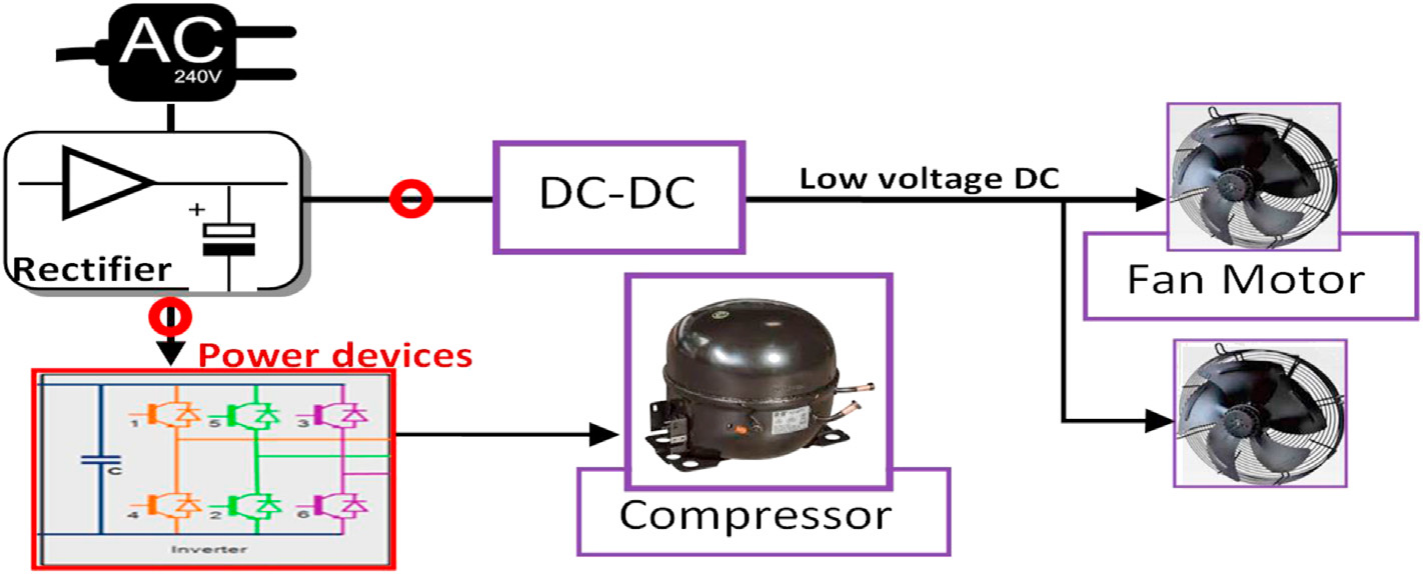
Block diagram of a refrigerator with a variable-speed compressor/motor.

In the same context, the new refrigerator with a variable-speed compressor/motor is demonstrated as a block diagram shown in Figure 7 [55].

The refrigerator capacity is (476 L), compressor type (Digital Inverter Compressor), 50Hz, 125W, and 230V. Since the power circuit of such a recent refrigerator has electronic elements that begin with a rectifier stage, the experimental measurements show the same performance under both AC and the proposed 310V DC power source in terms of power consumption. The inverter-driven refrigerator profile of power consumption shows a smooth starting as compared with the traditional one. The consumption rate rises due to the starting of the compressor running when its control tries to approach a set temperature. The power usually starts with half the maximum consumption and growing up gradually to a power rating value [55]. When the compartment temperature gets lower than a set value, the consumption gets down to its lower value, and continue fluctuating with some degradation and turned off unless no external action exists such as door opening.

#### 2.2.3 Motor-driven load (washing machines)

**Figure 8:**
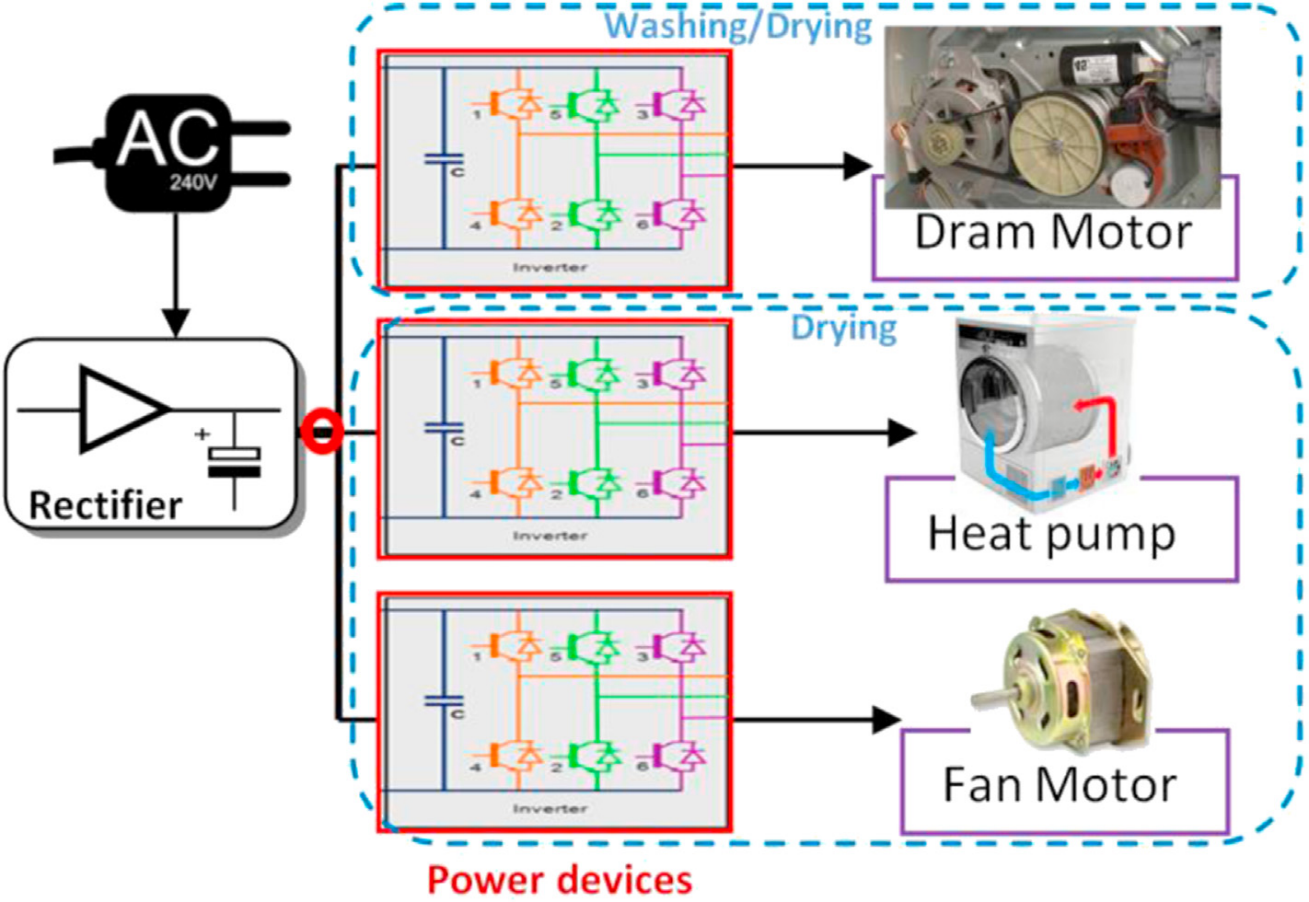
Block diagram of the speed-controlled motor-driven appliance (Washing Machine).

The mechanical parts of a traditional washing machine include an AC induction motor, which performs both tasks, spinning and washing with a single motor to reduce the weight and cost. Recently, an electronic drive is employed with a permanent magnet DC motor (PMDC) to deliver higher torque for the consumed power. This feature improves the energy efficiency of the machine and its linearity of torque-speed characteristics [56]. A general circuit diagram of an inverter-driven washing machine can be demonstrated in Figure 8, where the possible DC nodes are remarked by red circles.

## 3 Results

**Figure 9:**
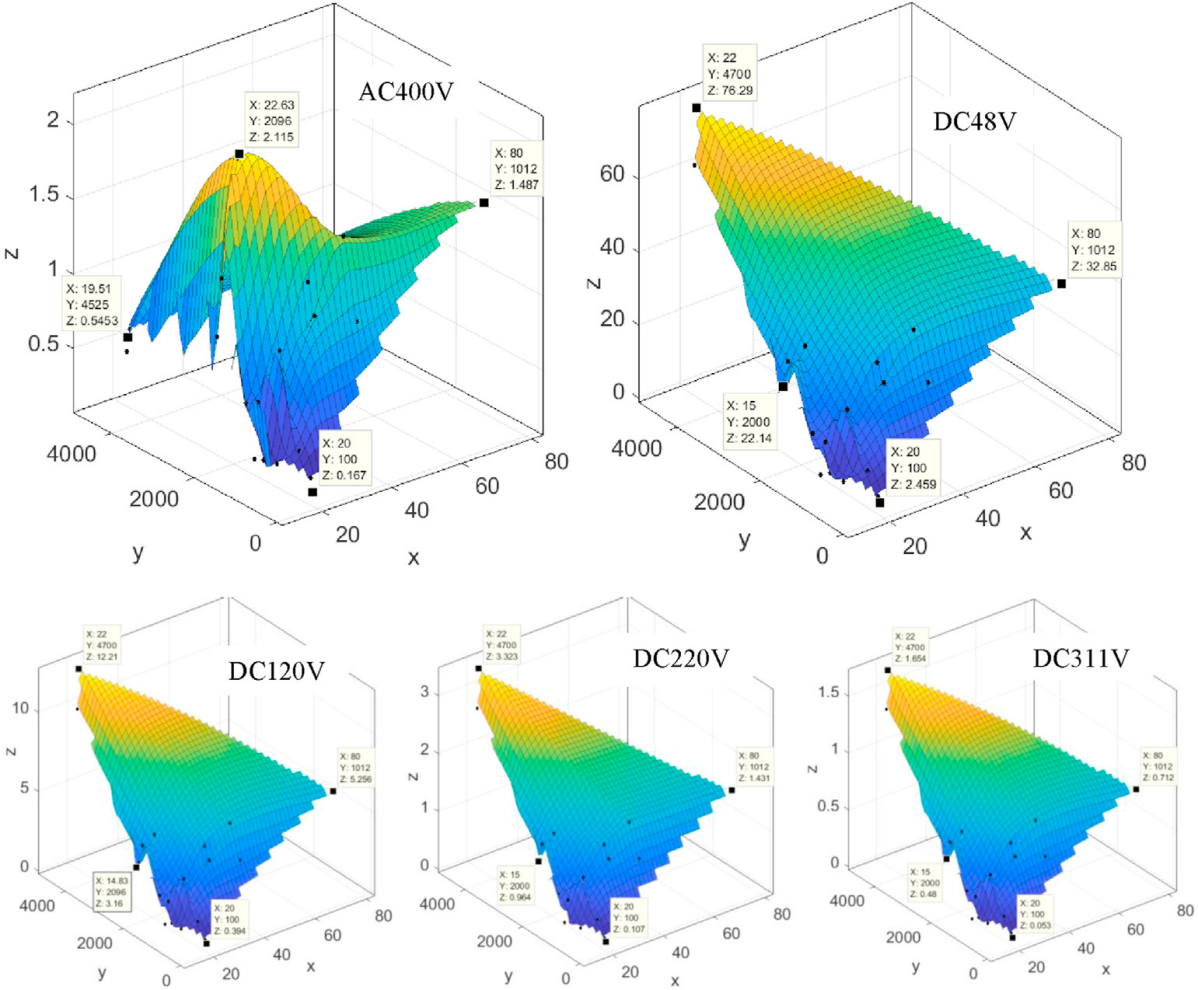
Power losses percentage (z-axis) along with the feeder (wire) length (x-axis) and the load power consumption (y-axis) when the source is 380V AC, 48V DC, 120V DC, 220V DC, and 311V DC respectively.

To verify the effectiveness of using DC source with the household appliances, experimental measurements have been conducted initially for the potential power losses. These measurements were taken as percentage values along with the feeder (wire) length and the load power consumption under both power sources, AC (380V line-to-line rated voltage, 3 phase) and DC with levels (48, 120, 220, 311)V [5]. The results are shown in Figure 9.

**Figure 10:**
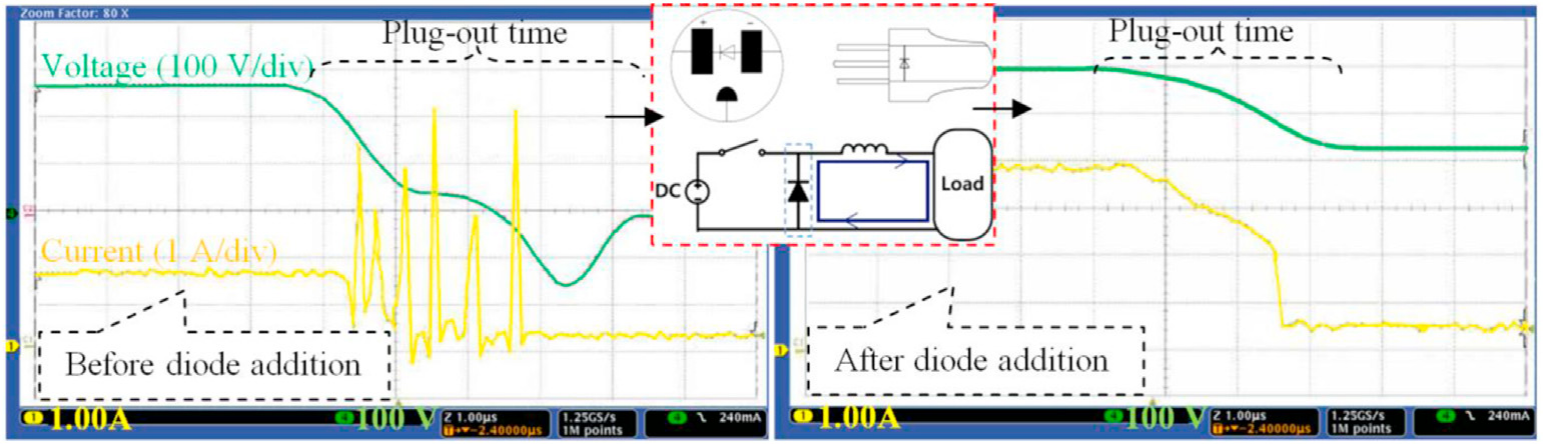
Oscilloscope capture current and a voltage waveform of the load at plug-out test under DC source, before (left) and after (right) adding a diode.

When using 48 V DC, both voltages drop, and power losses are too high. Also, the applicability of this voltage level to recent appliances with 1.5 and 2.5 mm^2^ cables has not existed. For the other voltage values (120, 230, 326 V dc), the results show that it is possible to substitute AC with DC and still use the same cables. The maximum voltage drop limit is not exceeded except for feeders when the load power exceeds 1.3KW in 120 V DC case. The power losses with 230 V DC are slightly lower than corresponding values for 230 V AC and the 311V DC is better. Therefore, this work proposes a topology with two voltage levels the 230V and 311V as a DC bus for all types of household appliances for PV-based with battery storage applications. The arc phenomena of the plugging-out duration of DC outlets can be reduced by a method using a freewheeling diode, which is verified experimentally as shown in Figure 10.

**Figure 11:**
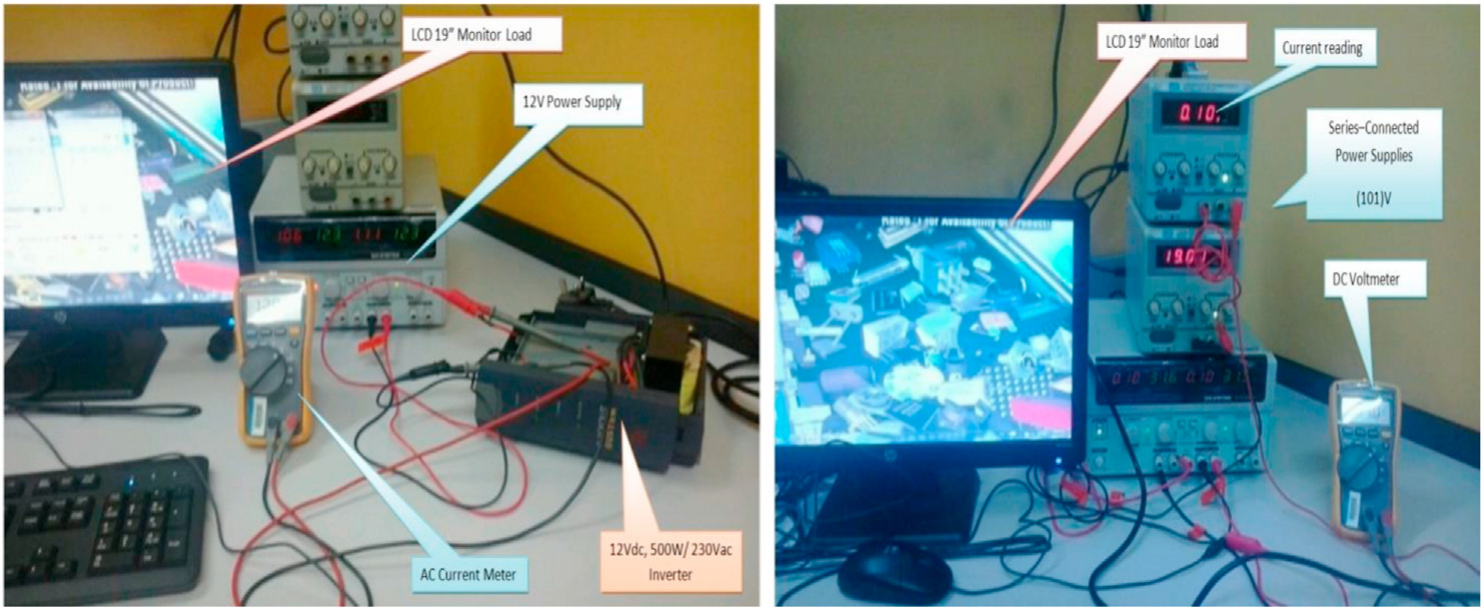
A 19″ LCD monitor in; (left) Source–Inverter–Load, (right) Source–Load configuration.

To validate the capability of DC power to drive SMPS-based electronic appliances, an experimental test has been implemented for a 19” LCD monitor. The obtained results have been compared with the corresponding results when the supply is AC. Figure 11 shows the experimental setup when performing the test on AC power source using a DC-AC inverter (on the left), and when applying the proposed connection with DC power source (on the right) directly. An inverter (12V input, 500W, 230V AC output) is used to feed power to the monitor, while a DC power supply (12V, 6 Amp) is used to supply the inverter. In contrast, the direct connection with the DC supply was conducted by setting the power supply to 101V that represents the proposed direct voltage matching configuration. The current, voltage power, and efficiency measurements are listed in Table 5.

**Table 5:** Experimental measurements of a 19” LCD monitor as an electronic appliance on DC and AC supply.

Parameters name	DC input	AC input
Supply voltage (V)	101	12
Supply current (A)	0.1	2.21
Load Voltage (V)	101	240
Load Current (A)	0.1	0.0424
LCD Consumption (W)	10.1	10.18
Inverter	No	Yes
Efficiency	99 %	10.18/(12∗2.21) = 38.38%

The above table shows that a significant change in inefficiencies when a direct source-load was used over the source-inverter-load configuration. This interprets the effectiveness of using DC power over AC power in battery-based PV home system applications.

**Figure 12:**
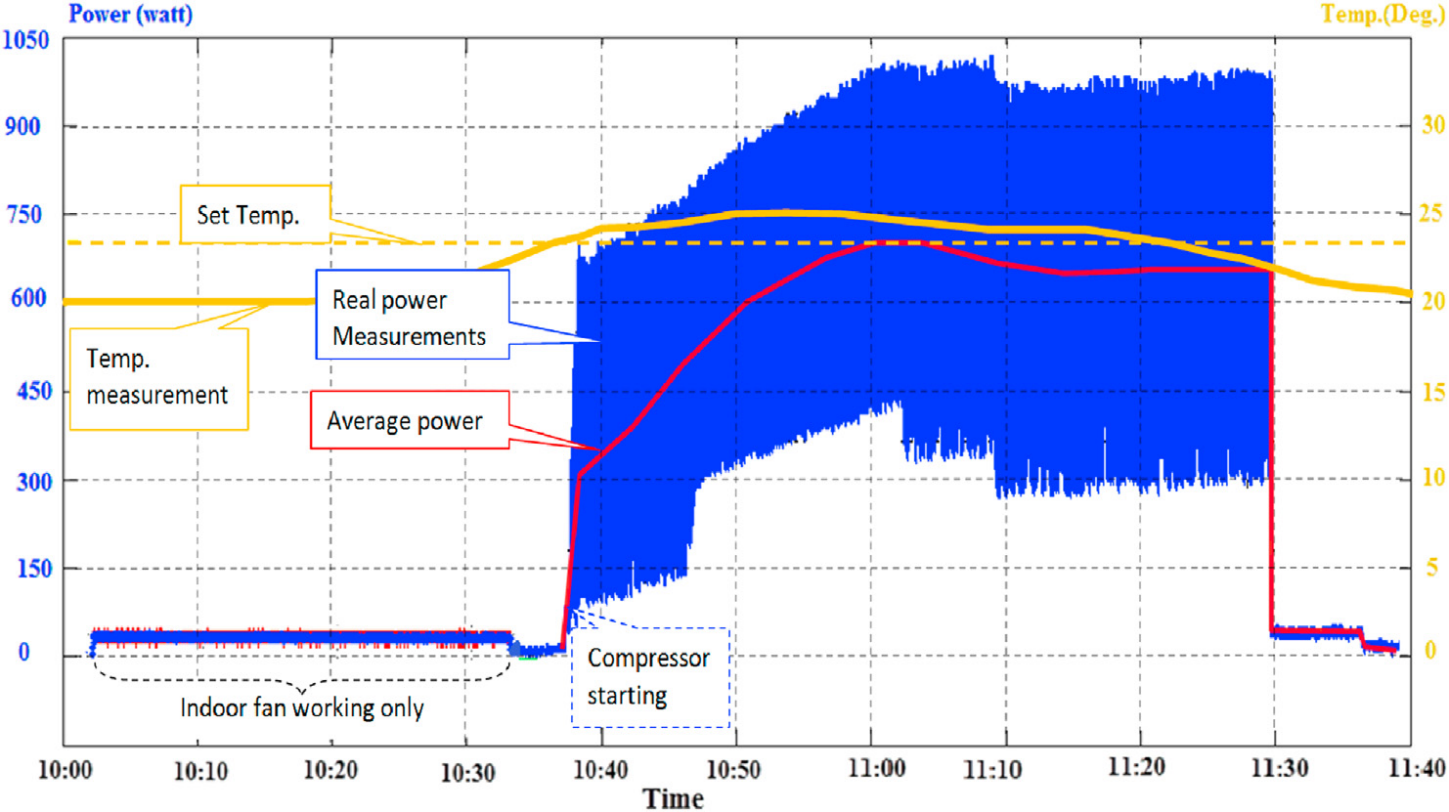
About one and half hour period, Experimental power consumption and temperature measurements for DC-bus supply on a 1 HP Air-conditioner.

About 1 h and a half of experimental measurements have been conducted that produced a power consumption waveform, as shown in Figure 12. The real measurements are shown with blue color while the average in red. Like the conventional air-conditioner, the consumption profile of the new inverter-based air-conditioner starts with the indoor fan and biasing power consumption of electronic elements for several minutes.

**Figure 13:**
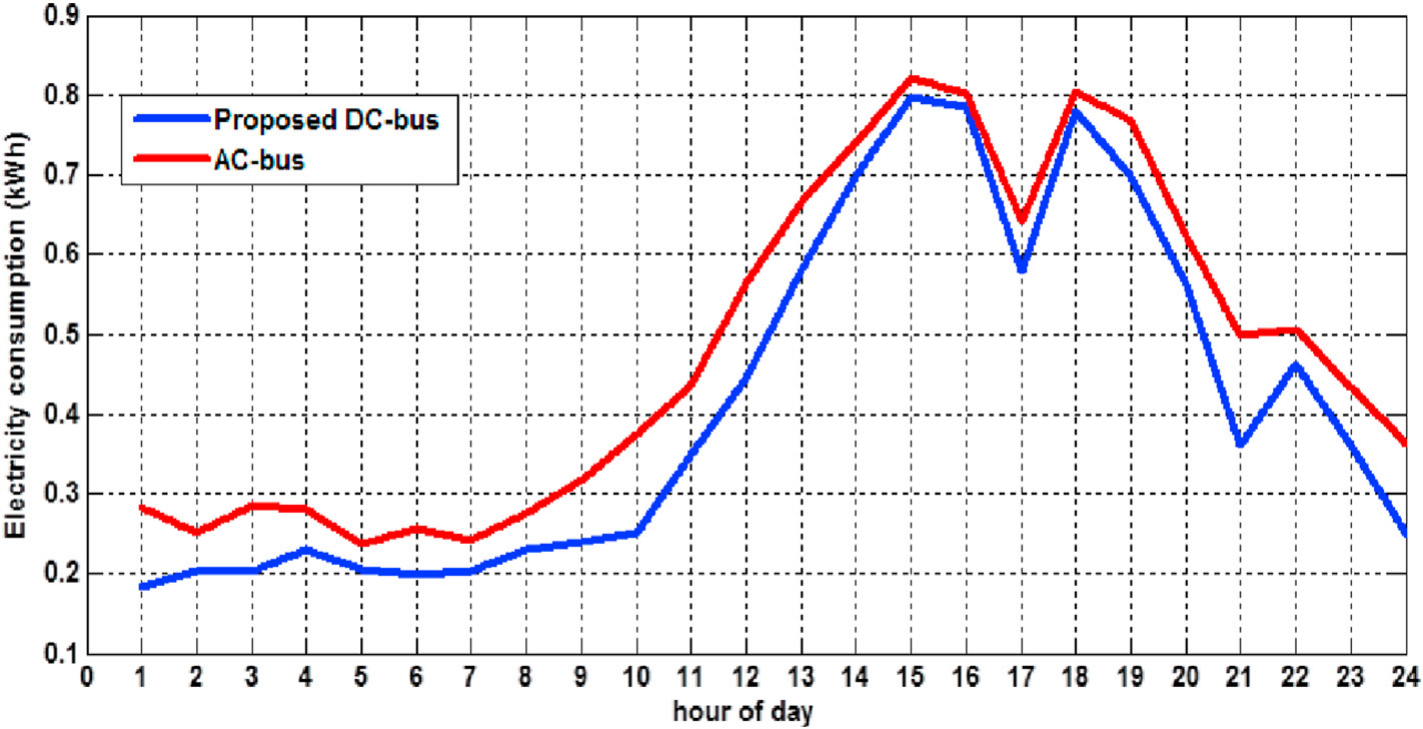
Air-conditioner energy consumption over a day.

An energy measurement has been carried out to test the power profile of inverter-driven air-conditioners under operating conditions, a normal AC power, and the proposed 310V DC supply, as shown in Figure 13. The results revealed that such air-conditioners consume a daily power up to 11.466 kWh, which includes the DC-AC power inverter for (DC source-inverter-load) configuration (red color). In contrast, consume daily power up to 9.850 kWh when directly coupled to the proposed (DC source-load) configuration (blue color).

From this experiment, It can be concluded that the curves of the power consumption under AC and DC tend to be converged when the average consumption becomes higher, which occurs at the noon period (14:00–19:00). This converges due to the rise of the inverter's efficiency when the power approaches (70–80) % of its maximum. The total daily energy is calculated by the integration of the area under the power curve over the daytime employing the Trapezoidal method. This result indicates a significant difference in energy consumptions between the traditional and proposed topologies, especially at lower levels of power.

## 4 Appliances comparison results

**Figure 14:**
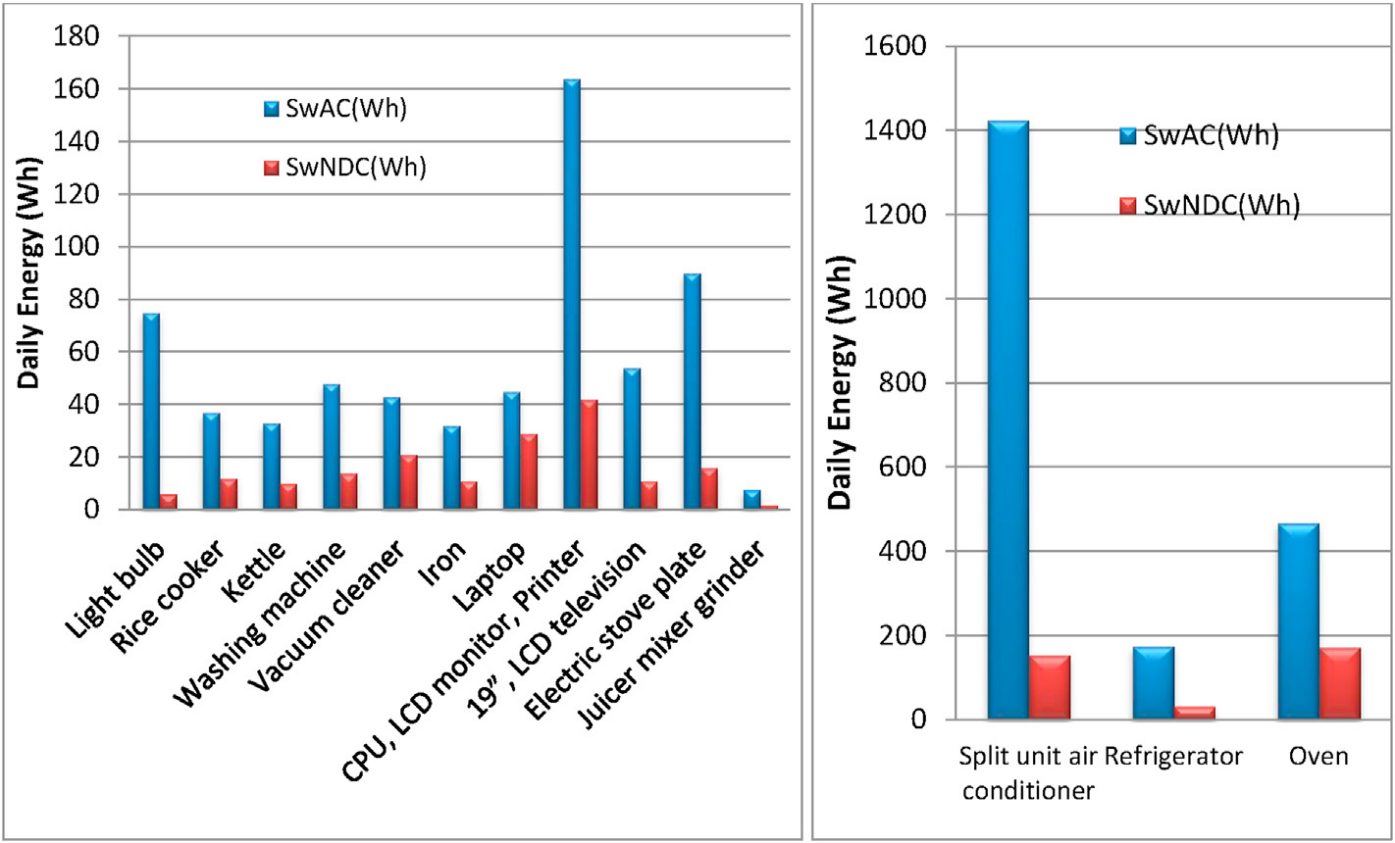
The daily energy savings with respect to traditional AC environment (SwAC), and with respect to the individually new DC environment in the literature (SwNDC).

To validate the effectiveness of the proposed configuration (DC source-appliances) over a traditional one (DC source-inverter-appliances), experimental measurements have been conducted for the most common appliances. Table 6 shows the comparison required parameters for energy evaluation when switching to the proposed system. The quantitative comparisons based on the savings that were harvested when switching to the proposed topology is shown in Figure 14.

**Table 6:** Voltages, rated power, and other parameters for energy evaluation measurements when switching to the proposed system.

Appliance	V	P	h/day	AC	NDC	PDC	SwAC	SwNDC
	(Vac)	(W)		(Wh)	(Wh)	(Wh)	(Wh)	(Wh)
Light bulb	220	60	12	797	728	722	75	6
Oven	220	800	1.5	1676	1380	1208	468	172
Rice cooker	220	500	0.75	412	387	375	37	12
Kettle	220	700	0.5	387	364	354	33	10
Washing machine	220	500	0.95	523	489	475	48	14
Vacuum cleaner	220	300	2	656	634	613	43	21
Iron	220	900	0.3	308	287	276	32	11
Refrigerator	220	125	13	1804	1661	1629	175	32
Split unit air conditioner	220	800	12	11466	9975	9850	1425	154
Laptop	220	50	7	398	382	353	45	29
CPU, LCD monitor, Printer	220	230	5	1320	1198	1156	164	42
19″, LCD television	220	90	5	508	465	454	54	11
Electric stove plate	220	850	1	943	869	853	90	16
Juicer mixer grinder	220	230	0.1	31.4	25.3	23.7	7.7	1.6
	Total Daily Savings (Wh)						2696.7	531.6

P is the rated power, h/day is the On-time hours/day, AC (Wh) is the AC-bus Daily energy (Wh), NDC(Wh) is the New DC-bus Daily energy (Wh), PDC(Wh) is the Proposed DC-bus matching Daily energy (Wh), SwAC is the Saving w.r.t AC-bus, SwNDC is the Saving w.r.t New DC-bus, and (w.r.t) denotes the (with respect to).

**Figure 15:**
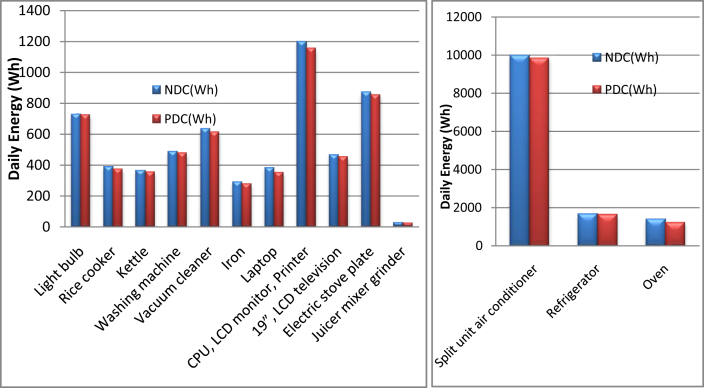
A comparison of the proposed DC microgrid (PDC) with the new DC operation in the literature (NDC) for the household appliances individually.

A comparison of the proposed DC microgrid (PDC) with the new DC operation in the literature (NDC) for the household appliances individually is also demonstrated as a bar chart in Figure 15.

It is found that the total daily energy saving for AC is 2696.7Wh, while it is 531.6Wh for NDC. Therefore the results verify the hypothesis of the work that is based on the voltage-matching concept in a DC system.

## 5 Conclusions and plans

Based on experimental measurements and our review for the household products, we conclude that it is possible now to provide a DC environment with all necessary appliances. This will allow the use of DC sources directly from onsite (battery storage, Photovoltaic), and to avoid the power losses due to converting DC to AC and back to DC. This work discussed the electrical diagrams of the recently available household appliances, and classified appliances into categories according to the way of consuming power. We proposed a new configuration based on the experiments and the related literature. The proposed topology reduced the losses of the overall system and grows its efficiency up. This modification was achieved by adopting a DC-environment with a battery bank that has only two levels of voltages to supply all user appliances.

### 5.1 Conclusions

1) It is found that the recent advances in household appliances direct towards DC compatible as appliances became including electronics in their power stage to drive and control their components. Therefore, all the appliances can be operated under a DC power source with an appropriate voltage level.
2) From an efficiency aspect, it is found that the available recent appliances (with DC-internally) are more efficient than the old AC counterparts. for some products, such as refrigerators, air-conditioners, the efficiency improvements can be around 20–30%.
3) The power losses with 230 V DC are slightly lower than corresponding values for 230 V AC and the 311V DC is better. Therefore, this work proposes a topology with two voltage levels the 230V and 311V as DC buses for all types of household appliances for PV-based with battery storage applications.
4) Using an appropriate freewheeling diode can significantly reduce the arc phenomena at the plugging-out duration of DC outlets.
5) The efficiency and reliability of a power distribution system can be better performed when decreasing the number of power conversion devices starting from the flow of power from sources (utility grid, solar PV, wind turbine, or on-site power generator) to consume by loads (household appliances).

### 5.2 Plans

a) It is essential to automatically select between the two DC voltage levels (${\text{V}}_{\text{FC}}$ and ${\text{V}}_{\text{FCR}}$) to recognize the resistive load.
b) A comprehensive analysis is still required to investigate the impacts of using the DC distribution system and its practical aspects. It is also required to increase the efforts for exporting the DC to a DC grid to facilitate the matching between the house microgrid and the utility grid.

## Declarations

### Author contribution statement

All authors listed have significantly contributed to the development and the writing of this article.

### Funding statement

This work was supported by 10.13039/501100008561Universiti Tenaga Nasional for the UNIIG fund (Project Code: J510050802).

### Data availability statement

Data included in article/supp. material/referenced in article.

### Declaration of interests statement

The authors declare no conflict of interest.

### Additional information

No additional information is available for this paper.
